# Multifunctional *Spirogyra-hyalina*-Mediated Barium Oxide Nanoparticles (BaONPs): Synthesis and Applications

**DOI:** 10.3390/molecules28176364

**Published:** 2023-08-31

**Authors:** Abdullah, Anees ur Rahman, Shah Faisal, Mervt M. Almostafa, Nancy S. Younis, Galal Yahya

**Affiliations:** 1 Department of Physical Chemistry and Technology of Polymers, Silesian University of Technology, M. Strzody 9, 44-100 Gliwice, Poland; 2 Joint Doctoral School, Silesian University of Technology, Akademicka 2A, 44-100 Gliwice, Poland; 3 Department of Health and Biological Sciences, Abasyn University, Peshawar 25000, Khyber Pakhtunkhwa, Pakistan; aneesurrah43@gmail.com; 4 Institute of Biotechnology and Microbiology, Bacha Khan University, Charsadda 24460, Khyber Pakhtunkhwa, Pakistan; shahfaisal11495@gmail.com; 5 Department of Chemistry, College of Science, King Faisal University, Alhofuf 31982, Al-Ahsa, Saudi Arabia; malmostafa@kfu.edu.sa; 6 Department of Pharmaceutical Sciences, College of Clinical Pharmacy, King Faisal University, Alhofuf 31982, Al-Ahsa, Saudi Arabia; nyounis@kfu.edu.sa; 7 Zagazig University Hospitals, Zagazig University, Zagazig 44519, Egypt; 8 Department of Microbiology and Immunology, Faculty of Pharmacy, Zagazig University, Al Sharqia 44519, Egypt; galalyehia@zu.edu.eg

**Keywords:** *Spirogyra hyalina*, nanoparticles, barium oxide, green synthesis, antioxidant, anti-inflammatory, antimicrobial

## Abstract

This research aims to biosynthesize Barium oxide nanoparticles (BaONPs) for biomedical applications, using *Spirogyra hyalina* as a stabilizing and reducing agent. UV–visible spectroscopy, Fourier transform infrared spectroscopy (FTIR), energy-dispersive X-ray, X-ray diffraction (XRD), and scanning electron microscopy (SEM) were used to physiochemically characterize the barium oxide nanoparticles, while antibacterial, minimum inhibitory concentration, antifungal, free radicle scavenging, and anti-inflammatory assay were performed to assess the therapeutic potential of the synthesized BaONPs. Fourier transform infrared spectroscopy revealed bands at 615 and 692 cm^−1^ that corresponded to the formation of BaONPs. Scanning electron microscopy revealed the spherical and flower-shaped morphology of BaONPs having an average diameter of 64.01 ± 2.0 nm. Both Gram-positive and Gram-negative bacterial growth was halted by the barium nanoparticles, demonstrating their efficacy up to 19.12 ± 0.31 mm against *E. coli*, 18.83 ± 0.44 mm against *Klebsiella pneumoniae*, 17.31 ± 0.59 mm against *P. aeruginosa*, 16.56 ± 0.37 mm against *S. aureus*, and 15.75 ± 0.38 mm against *S. epidermidis*, respectively. The minimum inhibitory concentration was 9.0, 6.3, 5.5, 4.5, and 2.0 µg/mL for *S. aureus*, *Klebsiella pneumoniae*, *S. epidermidis*, *P. aeruginosa*, and *E. coli*, respectively. BaONPs were not that effective against fungal strains such as *Rhizoctonia solani*, *Fusarium solani*, and *Fusarium proliferatum*. The BaONPs exhibited potent anti-inflammatory and antioxidant activity through inhibiting cyclooxygenases type 1 (43.12 ± 1.21%) and 2 (41.23 ± 1.56%), and DPPH free radicles up to 43.52 ± 0.29% at 400 µg/mL. In conclusion, the biomolecules derived from *Spirogyra hyalina* have demonstrated remarkable ability to generate stable nanoparticles, offering promising prospects for their utilization as therapeutic agents and coating materials in various biomedical applications.

## 1. Introduction

Synthesis of nanoparticles from biological sources such as algae is a new field of biotechnology known as “green synthesis”. A key step in the synthesis of nanoparticles is the reduction of metal ions, which may be accomplished by algae. Barium nanoparticles are renowned for their inhibitory effect on a wide range of bacteria and fungi [1,2,3,4]. Among these, BaONPs find diverse medical applications, including their integration into topical ointments and lotions as ionized compounds to combat infections [5]. Moreover, medical devices and implants are fortified against infections through the utilization of barium-coated polymers [6]. In the textile industry, innovative auxiliary equipment is emerging, encompassing barium sulfate-embedded polymers [7]. The synthesis of these nanoparticles can be achieved through diverse methods, encompassing both chemical and biological routes [8,9,10]. Notably, biological synthesis presents an eco-friendly avenue for generating barium oxide nanoparticles [1,2,3,4,5,6,7,8,9,10].

The utilization of biological molecules offers a substantial advantage in nanoparticle manufacturing, primarily due to their absence of hazardous chemically synthesized compounds. Moreover, they interact synergistically with naturally occurring capping agents [11]. The biological synthesis methodology has undergone meticulous refinement, with previous instances involving *Penicillium* spp., *Fusarium oxysporum*, and select bacterial strains [12,13,14]. Phytoextracts stand as a rich reservoir of metabolites essential for the stabilization and reduction of nanoparticles. Their widespread availability, ease of manipulation, and reliability have rendered them the preferred strategy for producing environmentally friendly, cost-effective nanoparticles [15]. This approach capitalizes on the efficient synthesis facilitated by the organisms’ abundant metabolite sources, making significant strides toward the production of steadfast nanomaterials by effectively reducing and capping metallic ions. A noteworthy example in this context is the green macroalga *Spirogyra* sp., characterized by its abundant presence of carbonyls, amino acids, and polyols. During the nanoparticle synthesis process, the biomolecules within *Spirogyra hyalina*’s extract serve as both reducing and stabilizing agents [16,17], attributed to the presence of alkaloids and flavonoids [11,12,13,14,15,16,17].

Given this backdrop, the prospect of synthesizing BaONPs from the extract of *Spirogyra hyalina* emerges as an intriguing proposition. The overarching objective of this study was to explore the potential of *Spirogyra-hyalina*-mediated biosynthesis, encompassing the comprehensive characterization and evaluation of barium oxide nanoparticles for their antibacterial, antifungal, anti-inflammatory, and antioxidant properties.

## 2. Results and Discussion

### 2.1. Extract Preparation and Nanoparticles Synthesis

**Figure 1:**
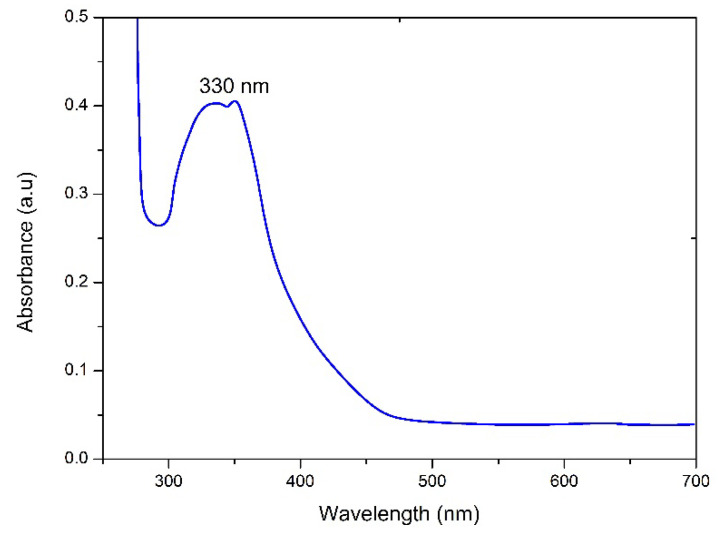
UV–visible spectroscopic analysis of BaONPs.

*Spirogyra hyalina* is ubiquitously found across various environments, spanning rivers, streams, and even small stagnant water bodies. Particularly thriving in limpid waters, *Spirogyra hyalina* thrives in the form of filamentous green masses that exude a slimy texture. A *Spirogyra* cell encompasses integral components such as a cell wall, nucleus, pyrenoid, and spiral chloroplasts. This species is distinctly rich in bioactive compounds including flavonoids, alkaloids, saponins, terpenoids, and amines [18,19,20]. Thus, the extract derived from *Spirogyra hyalina* emerges as a compelling biotemplate for the reduction of metal ions into nanoparticles. The preparation involved the amalgamation of algal extract and barium salt in a 1:1 ratio while maintaining temperatures conducive to preserving the structural integrity of algal biomolecules. A discernible shift in color from light to dark brown served as an early indicator of the successful production of nanoparticles. To ensure the proper reduction of barium ions into barium nanoparticles, the solution exhibiting the color change was left within a fume hood for a duration of 24 h [17]. Subsequent verification of the nanoparticles was accomplished through the utilization of a UV–vis spectrophotometer (UV-1602). This instrument facilitated an in-depth examination of the optical attributes of the synthesized barium oxide nanoparticles (BaONPs), with spectrum measurements spanning a range of 200 to 800 nanometers [17,18,19,20,21]. Surface plasmon resonance of barium nanoparticles peaked at around 330 nm, a characteristic signature consistent with BaONPs denoting successful synthesis of barium nanoparticles through this method [21], as shown in Figure 1. An alternate strategy for synthesizing BaONPs involves introducing a solution containing barium ions to *Spirogyra hyalina*. Algae inherently possess the ability to assimilate these ions from their environment. Upon internalization, these barium ions might undergo biomineralization processes within the algal cells, leading to the reduction of barium ions into nanoparticles. It is noteworthy that the distinct conditions and microenvironment within the cell could exert an influence on the resulting size and characteristics of the nanoparticles.

Subsequent to their generation, the BaONPs may potentially be released from the algal cells and diffuse into the surrounding liquid medium. However, this particular approach is still shrouded in limited understanding, and its effectiveness may yield a relatively scant quantity of nanoparticles. Furthermore, the intricate synthesis process demands specific temperature and environmental conditions, which might be challenging to sustain within the confines of an algal cell. Consequently, an in-depth and comprehensive investigation is imperative to ascertain the feasibility of successfully synthesizing nanoparticles through this approach [20,21].

### 2.2. FTIR and XRD Analysis of BaONPs

**Figure 2:**
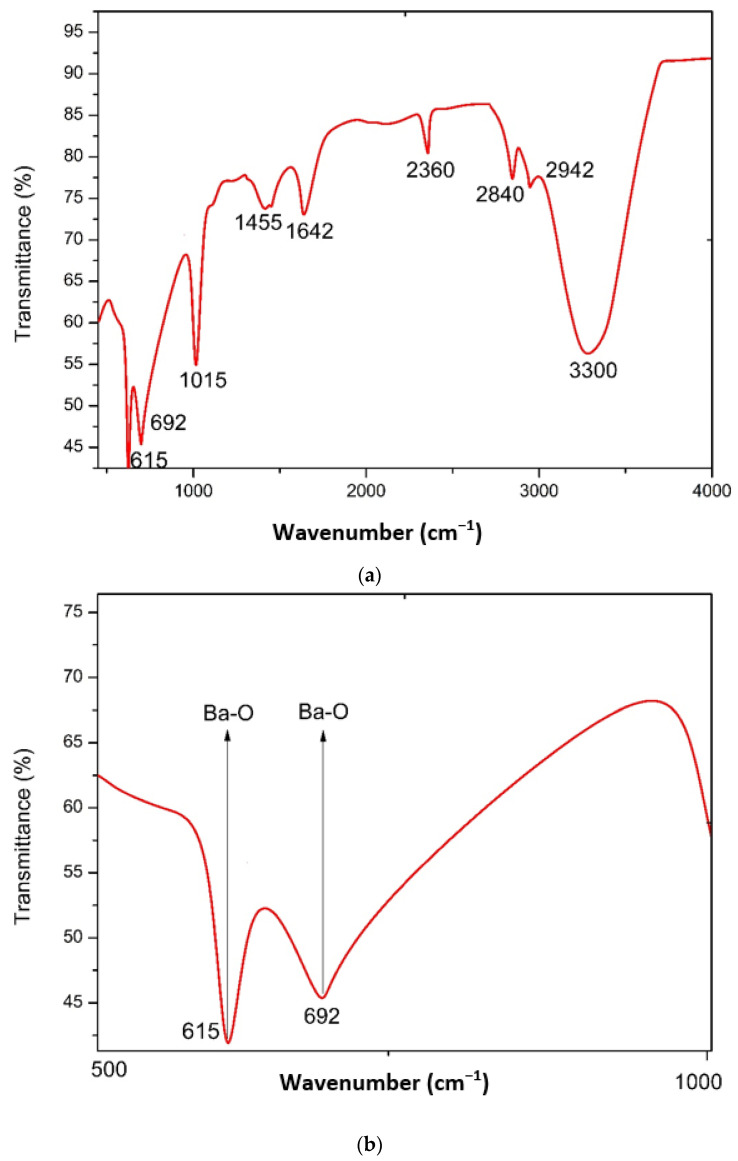
FTIR spectra of BaONPs (**a**), zoomed region of FTIR spectra identifying the BaONPs (**b**).

The FTIR technique was employed to investigate the chemical makeup and possible involvement of algal biomolecules in the reduction of barium to BaONPs throughout the wavenumber range of 4000–400 cm^−1^ [22], and the findings obtained are shown in Figure 2a,b. As a consequence of the surface adsorption of moisture and hydroxyl molecules, barium oxide nanoparticles may exhibit a wide absorption band between 3200 and 3600 cm^−1^. This band’s presence indicates the presence of hydroxyl groups (OH), which in turn leads to the existence of barium oxide nanoparticles. Vibrations and stretching in the metal–oxygen (Ba–O) bond may be the cause of an extra range of absorption that falls between 400 and 700 cm^−1^.

The synthesis of BaONPs was confirmed by the appeared band at 615 cm^−1^ that corresponds to strong stretching of the Ba–O bond [21]. At 692 cm^−1^, another band appeared for Ba–O. These bands confirmed the successful synthesis of barium oxide nanoparticles [21]. The bands at 1015, 1455, 1642, 2360, 2840, 2942, and 3300 cm^−1^ were assigned to stretching in C-N stretching in amines, medium C-H bending in alkanes, C=O stretching in amides, O=C=O stretching in atmospheric carbon dioxide [23]; alkanes exhibit C-H stretching, while carboxylic acids exhibit O-H stretching, as shown in Figure 2a,b. These functional groups might be due to the involvement of algal biomolecules in the formation of BaONPs [18]. It is essential to keep in mind that the unique FTIR spectrum of barium oxide nanoparticles may change based on a number of parameters, including the nanoparticles’ size, shape, surface functionalization, and the process used to synthesize them. When interpreting the FTIR spectrum, these factors need to be taken into account, and it is possible that a comparison with relevant reference materials is necessary.

**Figure 3:**
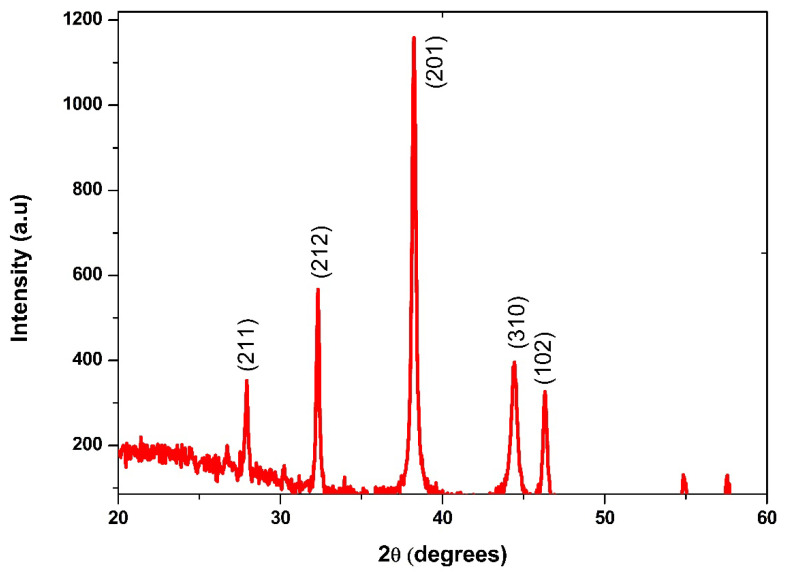
XRD analysis of BaONPs.

Biosynthesized BaONPs’ crystalline structure was analyzed by X-ray diffraction. The XRD spectra of the biosynthetically made BaONPs are shown in Figure 3. There are a few possible crystal structures for barium oxide, the most common of which are the cubic and hexagonal forms. In order to identify the crystal phase of the nanoparticles, the peaks from the XRD analysis may have their matched peaks compared to reference data for already-established crystal structures. Different planes of BaONPs, such as (211), (201), (102), (212), and (310), are reflected in the XRD pattern as distinct peaks. All these peaks line up perfectly with the tetragonal phase of BaONPs, and they are in perfect agreement with card No. 26-0178 from the “JCPDS” database. The outcomes that were found are also corroborated by the literature that was provided [21,24,25]. The fact that the manufactured nanoparticles have sharp and strong peaks is evidence that they are extremely crystalline in their natural state [26]. The average crystallite size (D) was calculated using the formula devised by Debye and Scherrer: *D = kλ/βCosθ* [27]. The average crystallized size of the samples was approximately ~40 ± 3.0 nm. By evaluating the strength of the diffraction peaks, it is feasible to gain some insight into the crystallinity of the nanoparticles. Peak intensities that are lower than average suggest the presence of amorphous aggregates, while peak intensities that are higher than average suggest that the sample has a greater degree of crystallinity. If there are impurities or secondary phases present in the sample, the XRD pattern may display additional diffraction peaks. These peaks may also be seen in the pattern after 50 degrees.

### 2.3. SEM and EDX Analysis

**Figure 4:**
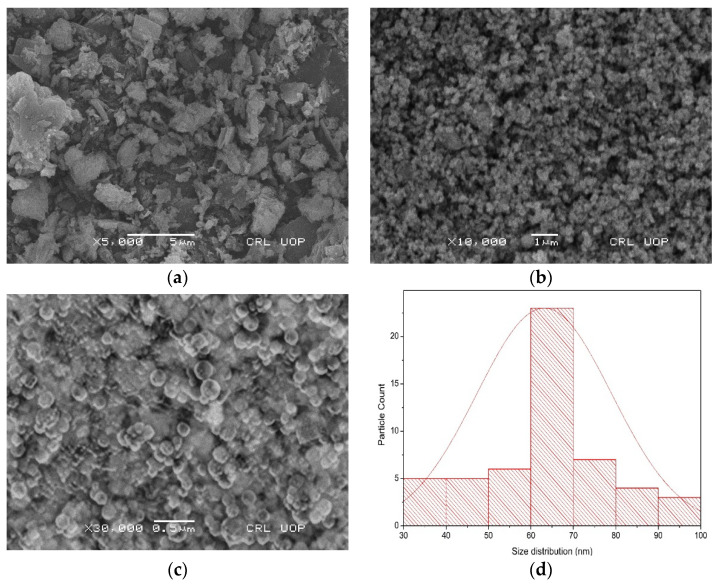
SEM images of magnification of BaONPs at ×5000 (**a**), ×10,000 (**b**), and ×30,000 (**c**). Size distribution graph of barium oxide nanoparticles (**d**).

Scanning electron microscopy was used to examine the morphology, and the elemental composition of the biosynthesized barium oxide nanoparticles was determined using energy-dispersive X-ray analysis in conjunction with scanning electron microscopy (SEM). As can be observed in Figure 4b, the form of the clusters is like amorphous aggregates [21], but at higher magnification, it seems more or less spherical, as shown in Figure 4c. A picture using a high-resolution SEM verified that the nanomaterials that were produced grew in a highly crystalline form. The SEM pictures also make it possible to observe that certain NPs are arranged in structures resembling nanosheets and are connected to one another by aggregation on top of one another having a size of more or less a micron, as shown in Figure 4a. According to ImageJ analysis, the average particle size was 64.01 ± 2.0; size distribution Figure 4d demonstrates that the particles were disrupted between 30 and 100 nm. The minimum particle size was found to be 36.65 ± 1.0, while the maximum was 93.84 ± 2.0. Chen et al. [28] researched the potential of extracts from four distinct produce items as building blocks for BaONPs. The synthesized BaSO_4_ NPs using kiwi fruits extract were spherical in shape, having a diameter of 2–4 μm, while those from tomatoes, oranges, and carrots were around 100 nm in size and rodlike or quasi-spherical in form. The formation mechanism of BaONPs showed that these four types of extracts containing organic compounds, proteins, vitamins, and carbohydrates were responsible for various morphologies of NPs.

**Figure 5:**
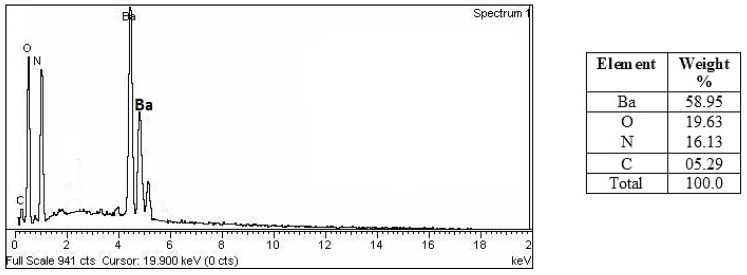
EDX analysis of the BaONPs.

Figure 5 displays the results of an EDX study showing distinct peaks for barium at 4.2, 4.4, and 4.6 KeV, with a weight percentage of 58.95. Oxygen, nitrogen, and carbon all showed up as separate peaks, and the weight percentages were 19.63, 16.13, and 5.29, respectively. The additional peaks indicated that algal biomolecules took part in the reduction [17,29] of barium ions to BaONPs.

### 2.4. Antibacterial Analysis

**Figure 6:**
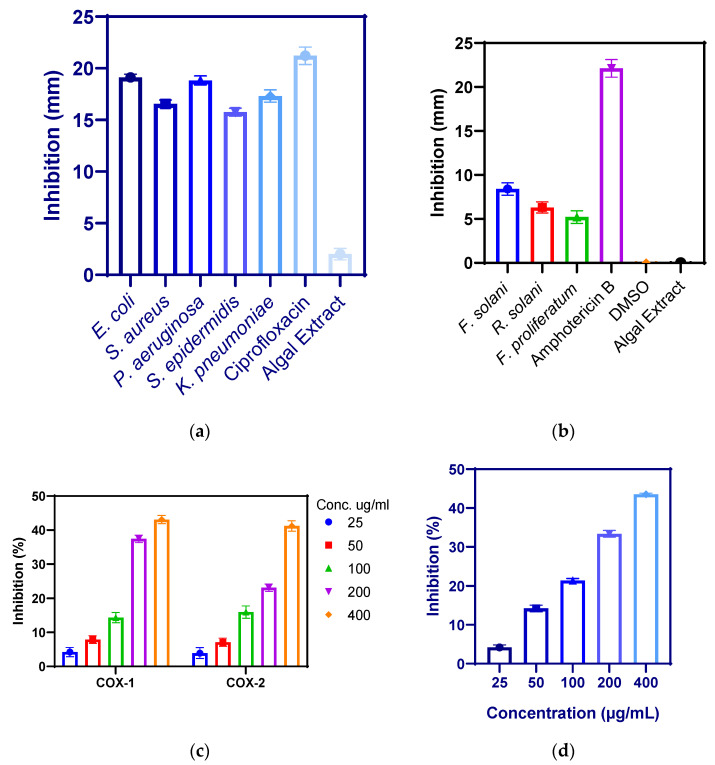
Antibacterial (**a**), antifungal (**b**), anti-inflammatory (**c**), and antioxidant activity (**d**) of barium nanoparticles.

The biosynthesized BaONPs showed antibacterial activity of 19.12 ± 0.31, 18.83 ± 0.44, 17.31 ± 0.59, 16.56 ± 0.37, and 15.75 ± 0.38 mm against *E. coli*, *P. aeruginosa*, *Klebsiella pneumoniae*, *S. aureus*, and *S*. *epidermidis*, respectively, as shown in Figure 6a. Antibacterial efficacy was greatest against *Escherichia coli* and lowest against *Staphylococcus epidermidis*; the minimum inhibitory concentration was observed for *S. aureus* while the least for *E. coli*, and almost similar results were found by [30]. The minimum Inhibitory concentration was 9.0, 6.3, 5.5, 4.5, and 2.0 µg/mL for *S. aureus*, *Klebsiella pneumoniae*, *S. epidermidis*, *P. aeruginosa*, and *E. coli*, respectively, as shown in Table 1. Similar results were found by [31]; *P. aeruginosa* and *S. aureus* exhibited significant sensitivity against BaONPs. The identification of cell membrane proteins in the extracellular matrix has previously shown that most nanoparticles target the cell membrane of the bacterium [17]. Sivakumar et al. [32] conducted a study in which they synthesized barium nanoparticles using the chemical precipitation method. The antibacterial activity suggests that the particles may interfere with Gram-positive and Gram-negative bacterial transporter, dehydrogenase, and periplasmic enzymatic activities. Sooch et al. designed a study to synthesize barium nanoparticles using gelatin as a capping agent. They doped the NPs with four metals to boost their physicochemical and antibacterial capabilities. These doped NPs have shown enhanced structural characteristics and antibacterial efficacy when compared to their bulk counterparts [33]. There have been a few experiments with barium nanoparticles (NPs) production in pharmaceutical and biological settings [34]. Upon adhering to the outer covering of bacteria, barium nanoparticles reduce metabolic pathways by obstructing cell wall permeability [35]. Biogenic NPs go deep into the cells, react with protein and DNA, and harbor biological harm to bacterial cells. NPs bactericidal activities are due to a large influx of ions from metallic particles that are known to have antibacterial characteristics [36,37,38,39,40,41,42,43]. Similarly, the size of NPs influences the degree of antibacterial effects. As a result, since smaller and minor particles are filled with the plentiful and even barium mass material, they demonstrate stronger antibacterial activities.

### 2.5. Antifungal Activity

The antifungal potential of biosynthesized nanoparticles was investigated by dissolving 1 mg of barium oxide nanoparticles in 1 mL of dimethyl sulfoxide (DMSO). The volume of 100 µL of nanoparticles was supplied to the wells that had previously been formed on sterile PDA plates that had been inoculated with fungal strains. As shown in Figure 6b, zones of inhibition were seen against (8.4 ± 0.7 mm against *Fusarium solani*), (6.30 ± 0.63 mm against *Rhizoctonia solani*), and (5.21 ± 0.72 mm against *Fusarium proliferatum*). The activity was performed three times, and the averages of the results were used to determine the real inhibitory zones. The barium nanoparticles did not show any significant activity against these fungal strains.

### 2.6. Anti-Inflammatory Assay

Substances or agents capable of reducing inflammation are considered anti-inflammatory [44]. Anti-inflammatory agents relieve severe inflammatory symptoms without affecting the CNS. Prostaglandin is produced by the enzyme’s cyclooxygenase types 1 and 2 [45]. At the location of an infection, inflammation is caused by the production of prostaglandins, which cause swelling, pain, redness, and fever. When these symptoms become more severe, they have the potential to disrupt the regular operations of the body. Because of this, inhibiting cyclooxygenases may bring about a reduction in inflammation. Significant results were observed for BaONPs by inhibiting the activity of COX-1 up to 43.12 ± 1.21% at 400 µg/mL, 37.42 ± 1.10% at 200 µg/mL, 14.36 ± 1.51% at 100 µg/mL, 7.91 ± 1.13% at 50 µg/mL, and 4.21 ± 1.37% at 25 µg/mL. BaONPs inhibited COX-2 up to 41.23 ± 1.56% at 400 µg/mL, 23.13 ± 1.11% at 200 µg/mL, 15.97 ± 1.81% at 100 µg/mL, 7.11 ± 1.19% at 50 µg/mL, and 3.91 ± 1.62% at 25 µg/mL, as shown in Figure 6c. On the other hand, the inhibition was found to be proportional to the dose, and it grew more pronounced as the number of NPs present in the solution increased [46]. In a previously reported study, Majumdar et al. observed that barium-doped bioactive glass (BaBG) within the nanoscale range has potent biocatalytic activity and inflammatory activity. BaBG was found effective in increasing IL-10, and as a result, it demonstrated anti-inflammatory properties [47]. Interleukin-10 is an anti-inflammatory cytokine that plays an important part in the prevention of autoimmune disorders as well as inflammatory diseases. Polymer-doped barium titanate nanoparticles have significant anti-inflammatory activity in bone regeneration [48]. The coating of barium nanoparticles with polymers and calcium magnesium ions can improve their anti-inflammatory activity.

### 2.7. Antioxidant Assay

The reactive oxygen species superoxide radicals, hydrogen peroxide, and hydroxyl radicals may all be scavenged by nanoparticles. This effect is brought about by the presence of functional groups on the NPs’ surface [49,50]. For the purpose of determining whether barium nanoparticles have an antioxidant effect, DPPH free radicals were subjected to test samples of varied quantities. BaONPs scavenged DPPH free radicles up to 43.52 ± 0.29% at 400 µg/mL, 33.37 ± 0.85% at 200 µg/mL, 21.41 ± 0.48% at 100 µg/mL, 14.21 ± 0.85% at 50 µg/mL, and 4.19 ± 0.61% at 25 µg/mL, as shown in Figure 6d. The amount of barium NPs proven to have an antioxidant effect was shown to be dosage-dependent. According to the findings, increasing the concentration of the nanoparticles led to a rise in the level of activity. The antioxidative activity of barium titanate (BaTiO_3_) nanoparticles did not have any effect on the generation of ROS in PC12 neural cell line [51]. Due to the presence of many components, including alkaloids, flavonoids, and others, barium oxide nanoparticles synthesized from *Linum usitatissimum* were able to boost the antioxidant activities [52]. These results suggested that biological biomolecules can improve the antioxidant activities of nanoparticles.

The green synthesis of BaONPs-mediated *Spirogyra hyalina* offers an efficient avenue for potential applications across various sectors. This approach underscores the innovative potential of metallic nanoparticle production, paving the way for the advancement of unique technologies [53]. The present study ventures into exploring alternative avenues for combating infectious diseases, shedding light on the utilization of biologically derived agents for the reduction and capping of nanomaterials. By doing so, we aim to spotlight the burgeoning trend of using nanomaterials for therapeutic purposes and to encourage the exploration of diverse natural sources for nanomaterial synthesis. This study contributes to the broader field of nanotechnology, an interdisciplinary pursuit focused on biochemistry applications, which seeks to develop nanoparticles with heightened antioxidant and antibacterial properties targeting degenerative diseases, cancer, and tumors [50,53].

This bioinspired method of nanoparticle green synthesis offers several advantages, including mild reaction conditions, eco-friendly fabrication, and the ability to generate nanoparticles with distinct characteristics. Through rigorous investigation, these nanoparticles can potentially evolve into impactful therapeutic agents with a wide array of applications, contributing significantly to the advancement of medical science and technology. 

## 3. Experimental

### 3.1. Spirogyra Hyaline Extract Preparation and Nanoparticles Synthesis

*Spirogyra hyalina* was collected from a local pond situated in Peshawar, Pakistan, and confirmed by the experts in the Department of Life Sciences, Abasyn University Peshawar, Pakistan. To prepare the extract, the algae was shade-dried and ground into powder, then we boiled 50 g of dry powder in 100 mL of dH_2_O for 30 min at 60 °C [17]. Once the liquid had cooled to room temperature after boiling, ultrafiltering was performed using Whatman filter paper No. 1. To obtain an extract that was both consistent and devoid of particles, the filtrate was centrifuged at 12,000 rpm. The supernatant of a greenish hue was separated from the pellet and placed in its own tubes. The extract was kept at 7 °C until it was time to make the NPs.

For the preparation of barium oxide nanoparticles, 50 mL of algal extract was mixed with 50 mL of 1.0 mM stock solution of barium nitrate (Ba(NO_3_)_2_) (Sigma Aldrich, Frankfurt, Germany, 99%) at room temperature and neutral pH, the mixing ratio was 1:1. The mixture was subjected to heat on a hot plate for 1 h at a temperature of 60 °C with continuous stirring. The solution was centrifuged at 12,000 rpm for 20 min and then dried in an oven at 80 °C to achieve pure nanoparticles [54]. After grinding, the nanoparticles were kept at 7 °C for further use.

### 3.2. Characterization of BaONPs

Spectral measurements between 200 nm and 800 nm were taken using a UV–vis spectrophotometer (UV-1602) to assess the BaONPs’ optical characteristics. Using a scanning electron microscope (JSM-JAPAN), we analyzed the morphological features of the synthetic BaONPs. An FTIR spectrometer (II), manufactured by Perkin Elmer, was used to examine the BaONPs’ chemical composition between the wavelengths of 400 and 4000 cm^−1^. The crystalline structure of BaONPs was verified by obtaining XRD data using a PANalytical X’Pert X-ray diffractometer. The elemental makeup of biosynthesized BaONPs was determined with the use of an EDS X Sight Oxford EDX analyzer [55,56,57].

### 3.3. Collection and Preparation of Bacterial Inoculum

All the biological activities were performed at Microbiology Research Laboratory Abasyn University, Peshawar, Pakistan. Gram-positive (*Staphylococcus aureus*, and *Staphylococcus epidermidis*) and Gram-negative (*Escherichia coli*, *Pseudomonas aeruginosa*, and *Klebsiella pneumoniae*) bacteria were included in the sample set. These bacteria were collected from the Hayatabad Medical Complex, Peshawar, and the Abasyn Microbiology Research Laboratory microorganism collection. These species were preidentified. Bacterial inocula were prepared by taking a visible colony of selected bacteria from a nutrient agar plate and transferred into screw-cap glass tubes containing Lysogeny. After inoculation, the tubes spent 24 h in a 37 °C incubator. Inoculated tubes showed bacterial growth after being incubated. Turbidity of the overnight cultures was adjusted to the No. 0.5 McFarland Standard [58,59], according to CLSI (Clinical Laboratory and Standard Institute) guidelines.

### 3.4. Antibacterial Activity

The Kirby–Bauer well diffusion technique was used to test the NPs’ antibacterial efficacy against bacteria [60,61,62]. A bacterial lawn was made on a nutrient agar (Merck, Germany) plate. Using a sterile cork borer, we drilled a well into the medium and then added NPs from a stock solution of 100 µg/mL of DMSO (1%). The positive control was ciprofloxacin (10 µg), while the negative control was DMSO. For 24 h, the plates were kept at 37 °C. The inhibitory zone was then measured in millimeters. 

### 3.5. Minimum Inhibitory Concentration (MIC)

After 24 h of incubation, the minimum inhibitory concentration of an antimicrobial agent is the concentration at which no further bacterial growth is detectable [63,64,65]. The concentration of nanoparticles used for MICs’ determination ranged from 0.5 to 20 µg/mL. MICs were performed in a 96-well flat-bottom polystyrene plate, and each well of the plate was loaded with 80 µL of bacterial inoculum and 20 µL of NPs. After inoculation, the plates spent 24 h at 37 °C; after incubation, the optical density of each well was checked by a plate reader at 600 nm to determine MIC using Equation (1).

(1) $$MIC\%=\frac{{OD}_{Cotrolled\;well}-{OD}_{treated\;well}}{{OD}_{controlled\;well}-{OD}_{blank\;well}}\times 100$$

### 3.6. Antifungal Activity

The antifungal potential of biosynthesized nanoparticles was investigated against plant pathogens *Rhizoctonita solani*, *Fusarium solani*, *and Fusarium proliferatum.* Stock solutions of NPs were prepared at 1.0 mg/mL of DMSO (1%). According to the well diffusion method [60], media plates with wells of 5–6 mm in diameter were drilled, inoculated with fungi, and 100 µL of nanoparticles was added to each well. We incubated the plates at 28 °C for a whole day. Zones of inhibition were calculated after incubation. Positive and negative controls were amphotericin B and DMSO, respectively.

### 3.7. Antioxidant Activity

BaONPs’ antioxidant potential was measured using 2,2-diphenyl-1-picrylhydrazyl (DPPH) as a free radical [66,67]. Different concentrations of NPs were prepared (25, 50, 100, 200, and 400 µg/mL) to be used in the antioxidant assay against DPPH free radicals. Then, 180 μL of DPPH solution (4.8 mg/50 mL of methanol) was mixed with 20 μL of the experimental sample and was poured into each well of a titer plate, followed by incubation at 37 °C for 30 min. The absorbance was then measured at 517 nm using a COBAS microplate reader after a 30 min incubation at 37 °C. During the assay, ascorbic acid served in the capacity of a positive control, and the experiment was carried out three times. The following Equation (2) was used to determine the percentage of free radical scavenging activity (FRSA):

% FRSA= (1Abs/Abc) × 100(2)

Absorbance of the sample is denoted by Abs, and that of the control, by Abc.

### 3.8. Anti-Inflammatory Assay

The test employed reagents for cyclooxygenase-1 (COX-1) and cyclooxygenase-2 (COX-2) from a French-made Ovine kit (701050) to look into the potential anti-inflammatory effects of barium oxide nanoparticles [68,69,70,71]. Nanoparticles at concentrations of 50–400 μg/mL were used to inhibit the activities of COX-1 and COX-2. Tetramethyl-p-phenylene diamine was detected by measuring the absorbance at 590 nm in a 96-well microplate reader. We used 10 mM of ibuprofen as a standard positive control.

## 4. Conclusions

In conclusion, *Spirogyra hyalina* has emerged as a promising biotemplate and environmentally friendly reducing agent, offering a sustainable and economically viable approach to nanoparticle synthesis. The biosynthesized BaONPs have demonstrated robust antibacterial, antioxidant, and anti-inflammatory properties, positioning them as a compelling candidate for future therapeutic applications. BaONPs hold potential for exploration in diverse domains such as drug delivery systems, targeted therapies, imaging applications, antiviral activities, cytotoxic effects on cancer cells, and their capacity to serve as therapeutic agents against various cancer types. Additionally, their ability to stimulate cell proliferation and facilitate tissue regeneration presents avenues for further investigation. To ascertain the viability of *Spirogyra-hyalina*-mediated BaONPs as therapeutic agents, comprehensive research is imperative. This research should encompass the evaluation of their therapeutic efficacy, safety profile, biocompatibility, and pharmacokinetics. 

## Figures and Tables

**Table 1:** Antibacterial and MIC of BaONPs.

Bacteria	BaONPs (20 µg/mL)	
	Zone of Inhibition	MIC (ug/mL)
*E. coli*	19.12 ± 0.31	2.0
*S. aureus*	16.56 ± 0.37	9.0
*P. aeruginosa*	18.83 ± 0.44	4.5
*S. epidermidis*	15.75 ± 0.38	5.5
* Klebsiella pneumoniae *	17.31 ± 0.59	6.3

## Data Availability

All the data are available within the manuscript. Additional data will be provided upon request from the corresponding author.
